# Water, Sanitation, and Hygiene Services in Public Health-Care Facilities in Indonesia: Adoption of World Health Organization/United Nations Children’s Fund Service Ladders to National Data Sets for a Sustainable Development Goal Baseline Assessment

**DOI:** 10.4269/ajtmh.18-0044

**Published:** 2018-06-25

**Authors:** Mitsunori Odagiri, Cahyorini, Khadijah Azhar, Aidan A. Cronin, Yulian Gressando, Indah Hidayat, Widya Utami, Karina Widowati, Airin Roshita, Rooswanti Soeharno, Sonny P. Warouw, Ardhiantie

**Affiliations:** 1 Water, Hygiene, and Sanitation (WASH) Section, United Nations Children’s Fund (UNICEF) Indonesia, Jakarta, Indonesia;; 2 National Institute of Health Research and Development, Ministry of Health, Jakarta, Indonesia;; 3 Environmental Health Unit, Ministry of Health, Jakarta, Indonesia;; 4 Health Section, United Nations Children’s Fund (UNICEF) Indonesia, Jakarta, Indonesia;; 5 Nutrition Section, United Nations Children’s Fund (UNICEF) Indonesia, Jakarta, Indonesia;; 6 Community Health and Nutrition Unit, National Development Planning Agency (Bappenas), Jakarta, Indonesia

## Abstract

Provision of basic water, sanitation, and hygiene (WASH) services in health-care facilities is gaining increased attention, given growing acceptance of its importance to the maternal and newborn quality of care agenda and the universal health coverage framework. Adopting and contextualizing an emerging World Health Organization/United Nations Children’s Fund Joint Program Monitoring service ladder approach to national data collected in 2010/2011, we estimated the national coverage of primary health centers (PHCs) (*N* = 8,831), auxiliary PHCs (*N* = 22,853), village health posts (*N* = 28,692), and village maternity clinics (*N* = 14,396) with basic WASH services in Indonesia as part of a Sustainable Development Goal baseline assessment. One quarter of PHCs did not have access to a combination of basic water and sanitation (WatSan) services (23.6%) with significant regional variation (10.6–59.8%), whereas more than two-third of PHCs (72.0%) lacked handwashing facility with soap in all three locations (general consulting room, immunization room, and delivery room). More than a half of the three lower health service level facility types lacked basic WatSan services. National health facility monitoring systems need to be urgently strengthened for tracking the progress and addressing gaps in basic WASH services in health facilities in Indonesia.

The sustainable development goal (SDG) target six calls for the elimination of open defecation and universal access to drinking water, sanitation, and hygiene (WASH) that are safely managed services both at the household-level and in institutional settings, including schools, health-care facilities (HCFs), workplaces, and other public spaces.^1^ Provision of basic WASH services in HCFs is gaining increased attention in light of maternal newborn quality of care agenda, antimicrobial resistance control, and the universal health coverage (UHC) framework.^2,3^

In Indonesia, improving neonatal mortality and nutritional status among children remains challenging. The neonatal mortality rate is estimated to be 19 deaths per 1,000 live birth and has remained stagnant over the past 20 years.^4^ Indonesia still accounts for the eighth largest number of neonatal deaths in the world.^5^ One of the leading causes is sepsis, accounting for 12% and 21% of mortality of 0–6 days and 7–28 days age groups, respectively.^6^ Thirty-seven percent and 12% of children less than 5 years old are affected by stunting and wasting, respectively.^7^ Neonatal mortality and undernutrition can potentially be substantially reduced through basic WASH interventions such as handwashing with soap and clean birth practices in both homes and HCFs.^8^ Achieving universal access to WASH in Indonesia remains a major challenge with over 30 million people still practicing open defecation.^1^ Furthermore, there is currently no regular comprehensive national monitoring mechanism for WASH in HCFs.^9^

An assessment in 54 low- and middle-income countries (LMICs) provided a broader perspectives of poor WASH service provision in HCFs,^10^ whereas a recent comprehensive existing data analysis from 78 LMICs identified inequalities and environmental health challenges in HCFs.^11^ Importantly, both studies highlighted the need for further data collection, especially in Southeast Asia.^10,11^ There are limited journal-published studies on WASH in HCFs mostly in Africa,^12–14^ and rural Cambodia.^15^ Efforts to establish a SDG baseline for WASH in HCFs have been underway in several countries,^1^ but to our best knowledge, few studies have examined the condition of WASH in HCFs in the Southeast Asia region including Indonesia, except three WASH services in HCFs overview reports.^9,16,17^

Toward this end, we adopted and contextualized an emerging global framework for WASH in HCFs^18^ proposed by the World Health Organization (WHO)/United Nations Children’s Fund (UNICEF) Joint Monitoring Program (JMP) to existing national HCF census data sets and estimated basic WASH service coverage across four types of public HCFs in Indonesia as a SDG baseline assessment. In a secondary analysis, we explored disparities in basic WASH service in HCFs across the geographic regions of the country. We analyzed two national HCF census data sets, *Riset Fasilitas Kesehatan* (Rifaskes or health-care facility research) for primary health centers (PHCs) (*N* = 8,831), and *Potensi Desa* (village potential data) data set to look at auxiliary PHCs (*N* = 22,853), village health posts (*N* = 28,692), and village maternity clinics (*N* = 14,396). In this study, HCFs refer to these four types of facilities and do not include hospitals (*N* of public hospital = 685 in 2011), whereas PHCs refer to only PHCs, and does not include auxiliary PHCs. These data were collected by Ministry of Health (MoH) and the National Bureau of Statistics, respectively, in 2010 and 2011, based on observation by enumerators. Questionnaires are available online (See Supplemental Material).

Efforts were made to align standard definitions from existing surveys with the emerging global framework for each level of HCFs (Table 1). In addition, three combined indicators were constructed, which include 1) combined basic water and sanitation (WatSan), 2) combined basic WASH (in a general consulting room) (WatSan and Hygiene), and 3) combined basic water, sanitation, hygiene (in a general consulting room), and health-care waste management services (WatSan, Hygiene, and Waste), to assess efforts needed for providing comprehensive basic WASH services in HCFs.

**Table 1:** Definition of basic, limited, and no water, sanitation, hygiene and health-care waste services in the WHO/UNICEF JMP service ladder framework and this study

	Water		Sanitation		Hygiene		Health-care waste	
	WHO/UNICEF JMP framework	This study	WHO/UNICEF JMP framework	This study	WHO/UNICEF JMP framework	This study	WHO/UNICEF JMP framework	This study
Basic services	Water from an improved source is available on premises.	PHCs*: Water from piped systems, boreholes, wells, spring, or rainwater is available for all year-round.	Improved sanitation facilities are useable, separated for patients and staff, separated for women and allowing menstrual hygiene management, and meeting the needs of people with limited mobility.	PHCs: There is a toilet and water is available inside.	Hand hygiene materials, with a basin with water and soap or alcohol hand rub, are available at points of care and toilets.	PHCs: A hand hygiene station is available with soap in a general consultation room.	Waste is safely segregated into at least three bins in the consultation area and sharps and infectious wastes are treated and disposed of safely.	PHCs: Medical waste is separated and safely disposed of (i.e., with an incinerator)
		Other types of HCFs†: Water from piped systems, boreholes, wells, spring or rainwater that are on premises.		Other types of HCFs: There is a toilet for patient that is functional.		Other types of HCFs: Data not collected‡		Other types of HCFs: Data not collected‡
Limited services	Water from an improved source is available off-premises or an improved water source is on site but water is not available.	PHCs: Water from piped systems, boreholes, wells, spring or rainwater, but is not available for all year-round.	Improved sanitation facilities are present but are not usable, or do not meet the needs of specific group (staff, women, and people with limited mobility).	PHCs: There is a toile but water is not available inside.	Hand hygiene station at either point of care or toilet, but not both.	PHCs: Data not collected	Waste is segregated but not disposed of safely, or bins are in place but not used effectively.	PHCs: Medical waste is separated, but unsafely disposed of (i.e., burned or buried).
		Other types of HCFs: Water from piped systems, boreholes, wells, spring or rainwater that are off-premises.		Other types of HCFs: There is a toilet for patient, but not functional.		Other types of HCFs: Data not collected		Other types of HCFs: Data not collected
No services	Unprotected dug well or spring, surface water source or there is no water source at the facility	PHCs: Water from other source.	Pit latrines without a slab or platform, hanging latrines and bucket latrines, or there are no toilets or latrines at the facility	PHCs: There is no toilet at the facility.	Hand hygiene stations are absent or present but without soap or water	PHCs: Hand hygiene stations are absent or present but without soap in a general consultation room	Waste is not segregated or safely treated and disposed	PHCs: Medical waste is not separated.
		Other types of HCFs: No water available for patient bathroom, water from surface water source or there is no water source at the facility		Other types of HCFs: There is no toilet at the facility		Other types of HCFs: Data not collected		Other types of HCFs: Data not collected

HCFs = health-care facilities; JMP = joint program monitoring; WHO = World Health Organization; UNICEF = United Nations Children’s Fund.

* PHCs: Primary health centers.

† Other types of HCFs: Other types of health-care facilities include auxiliary PHCs, village health post, and village maternity clinic.

‡ Data not collected: Indicators of a handwashing station and medical waste practice in other three types of HCFs were not collected in Potensi Desa (village potential data).

The proportions of PHCs with access to basic WatSan were significantly higher than those of the other three types of HCFs (Figure 1 and Supplemental Table 1 in Supplemental Material). Most of PHCs had access to basic water (82%) or sanitation services (88%), whereas approximately just more than half of lower health service level HCFs did, suggesting levels of WatSan services vary significantly between PHCs and lower HCF types. Despite the relatively better conditions of WatSan facilities in PHCs, their hygiene and health-care waste management services were found to be poor (Figure 1). A handwashing station with soap was observed in more than half of general consulting rooms (54%) and delivery rooms (67%) but only in one-third of immunization rooms (33%). Only 28% of PHCs had a handwashing station with soap in all three locations. Just more than one-third of PHCs (35%) provided basic health-care waste management services. A recent study in Indonesia suggested improved conditions (87% of HCFs with at least one handwashing facility).^11^ The results, however, need to be compared with caution given that the data are subnational representative. Looking at combined WASH services, most of the PHCs had access to basic WatSan services (WatSan: 76%), which was significantly higher than the lower level HCFs (WatSan: 45–49%) (Table 2). However, most of PHCs were not observed to have all of basic water, sanitation, hygiene, and health-care waste services (WatSan, Hygiene, and Waste: 20%) (Table 2). A recent study of WASH in HCFs in rural regions of sub-Saharan Africa reported less than half of rural HCFs with access to combined basic WASH services.^12^ Despite slightly different definition, our study found similar levels of combined basic WASH access in rural PHCs (WatSan and Hygiene: 41%) (Supplemental Table 1 in Supplemental Material). Six LMICs’ nationally representative survey analysis demonstrated challenging results in looking at all four services; just 2% of HCFs provide all of water, sanitation, hygiene, and waste management services.^11^ In this study, the national coverage of PHCs with access to all four basic services was also significantly lower compared with the coverage of basic WatSan services (Table 2). Observed low levels of all four basic WASH service provisions in PHCs, particularly because of a lack of handwashing facilities, indicate an urgent need for actions as these conditions not only increase risks of hospital-acquired infections^19^ but also negatively impact on women’s health-seeking behavior around antenatal care and delivery at PHCs.^20^

**Figure 1.**
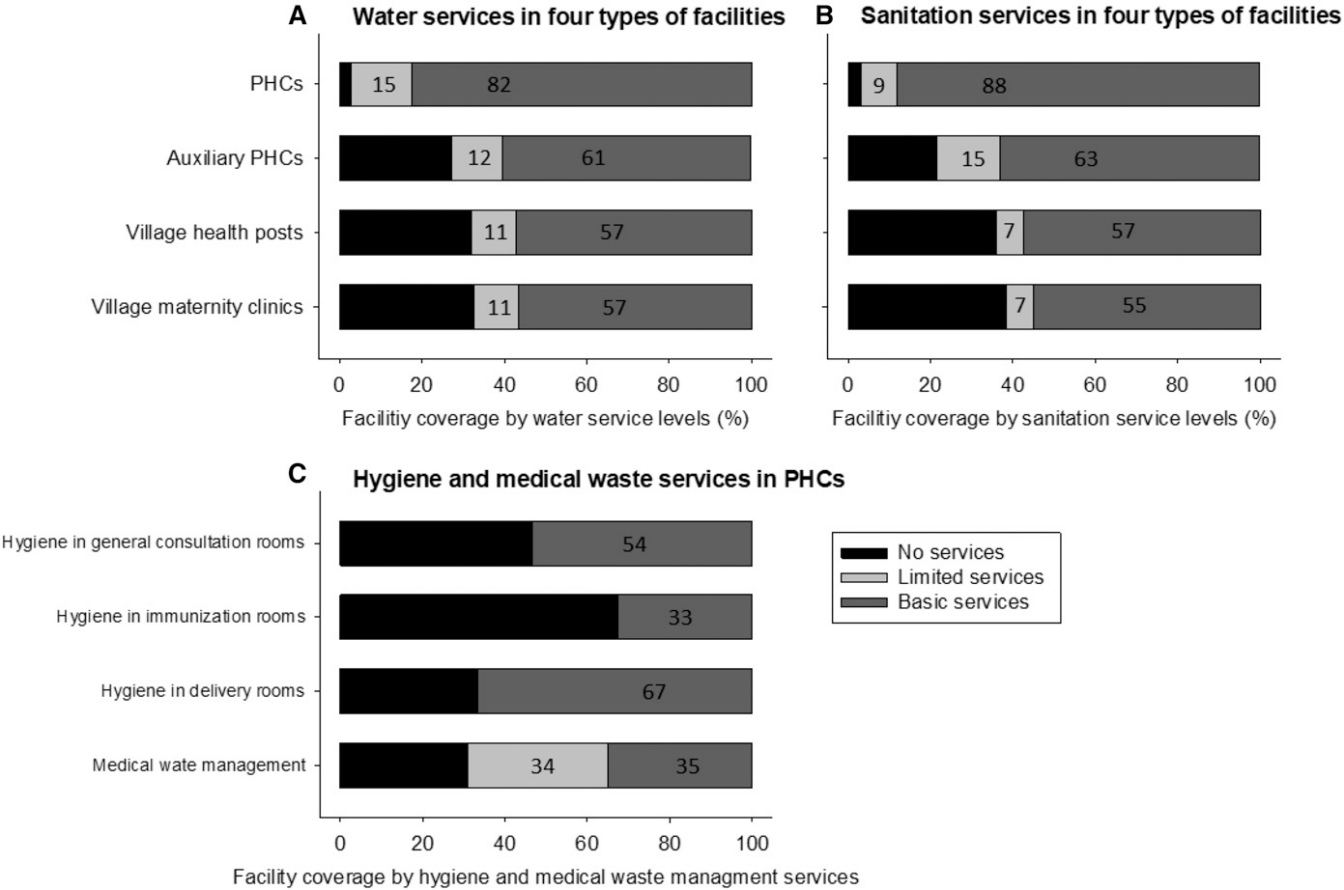
Coverage of basic, limited, and no services of (**A**) water and (**B**) sanitation in primary health centers (PHCs) (*N* = 8,831), auxiliary PHCs (*N* = 22,853), village health post (*N* = 28,692), and village maternity clinic (*N* = 14,396), (**C**) hygiene and health-care waste management in PHCs (general consultation rooms, *N* = 8,831, immunization rooms *N* = 5,628, delivery rooms = 3,097, and medical waste management *N* = 8,831). For hygiene service levels, because of a lack of information on handwashing stations in or near a toilet in PHCs, distinction between basic and limited services is not made. The number in bar charts indicates the proportion of each category in percentage.

**Table 2:** National, Region 1 (Java and Bali), and Region 4 (Maluku and Papua) coverage of combined indicators and percentage point difference between Region 1 and 4 in four types of public health-care facilities

Type of facilities (combined indicators)	National coverage (95% CI*)	Coverage in Region 1† (95% CI)	Coverage in Region 4‡ (95% CI)	Relative ratio of Region 1 to Region 4	Percentage point difference between Region 1 and Region 4 (95% CI)
PHCs§ (WatSan, Hygiene, and Waste‖)	20.2 (19.4–21.1)	36.5 (35.0–38.1)	2.5 (1.3–3.7)	14.6	34.0 (32.0–36.0)
PHCs (WatSan and Hygiene¶)	46.2 (45.1–47.2)	68.0 (66.5–69.6)	17.2 (14.3–20.1)	4.0	50.8 (47.5–54.2)
PHCs (WatSan#)	76.4 (75.5–77.3)	89.4 (88.4–90.4)	40.2 (36.4–44.0)	2.2	49.2 (45.2–53.1)
Auxiliary PHCs (WatSan)	49.1 (48.5–49.8)	63.0 (61.8–64.1)	32.0 (29.6–34.4)	2.0	30.9 (28.2–33.7)
Village health posts (WatSan)	46.2 (45.6–46.8)	49.2 (48.4–50.1)	22.4 (18.3–26.4)	2.2	26.9 (22.7–31.0)
Village maternity clinics (WatSan)	45.1 (44.3–45.9)	57.7 (56.5–58.9)	20.6 (17.3–23.8)	2.8	37.1 (33.6–40.6)

* 95% CI: 95% Confidence Interval.

† Region 1: Java and Bali (PHCs: *N* = 3,578, auxiliary PHCs: *N* = 6,631, village health posts: *N* = 9,701, village maternity clinics: *N* = 6,325).

‡ Region 4: Maluku and Papua (PHCs: *N* = 634, auxiliary PHCs: *N* = 1,471, village health posts: *N* = 407, village maternity clinics: *N* = 588).

§ PHCs = primary health centers.

‖ WatSan, Hygiene, and Waste: A combined indicator defined as a facility with access to basic water, sanitation, hygiene (in a general consultation room), and health-care waste management services.

¶ WatSan and Hygiene: A combined indicator defined as a facility with access to basic water, sanitation, and hygiene (in a general consultation room) services.

# WatSan: a combined indicator defined as a facility with access to basic water and sanitation services.

To examine disparities in basic WASH service access in HCFs between regions, we divided Indonesia into four regions: Region 1 (Java and Bali), Region 2 (Sumatra), Region 3 (Kalimantan, Sulawesi, Nusa Tenggara Barat, and Nusa Tenggara Timur), and Region 4 (Maluku and Papua), based on geographic proximity and household water and sanitation coverage, in consultation with government stakeholders. Across all four types of HCFs investigated, there was a consistent trend showing Region 1 (i.e., Java and Bali) with the highest basic WASH service coverage and Region 4 (i.e., Maluku and Papua) with the lowest (Figure 2). In the most challenging area, Region 4, most of all types of HCFs did not provide combined basic WatSan services (WatSan: 21–40%); these figures are significantly lower than the national average (Table 1). To quantitatively assess regional disparities between Region 1 and 4, we measured relative (i.e., the ratio of coverage of Region 1 to 4) and absolute (i.e., a percentage point difference between Region 1 and 4) disparities in basic WASH access (Table 2) as described elsewhere.^21^ The relative disparity appeared to be the greatest in combined basic water, sanitation, hygiene (in a general consulting room), and health-care waste management service (WatSan, Hygiene and Waste) coverage of PHCs. The greatest absolute disparity was observed in 1) combined basic WASH (in a general consulting room) service (WatSan and Hygiene) coverage in PHCs and 2) combined basic WatSan service coverage in PHCs (Table 2). Similarly, subnational level disparities of WASH service provision in HCFs were reported in Sierra Leone, Kenya, and Ethiopia.^10^ Furthermore, our additional analysis, looking at correlation between coverage of basic WASH (in a delivery room) services in PHCs and home birth rate^4^ at provincial-level, indicates provinces with higher home birth rates had PHCs with lower levels of WASH services (Figure 3). This underscores that WASH services, amongst other factors, need to be urgently improved for a safe delivery while promoting a delivery in HCFs. These findings underline the need for greater efforts to improve WASH conditions in both households and HCFs in unserved regions and communities as highlighted in SDGs.^1^

**Figure 2.**
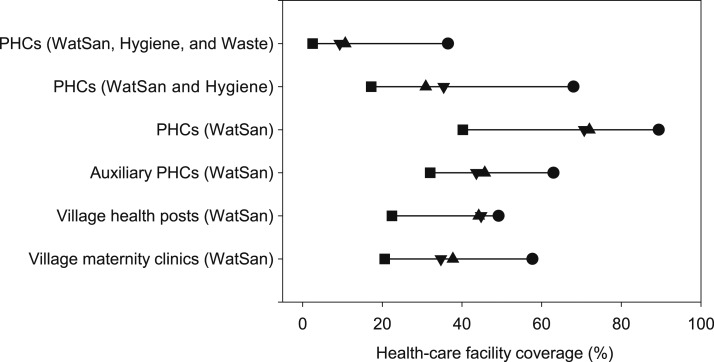
Four types of public health-care facility coverage of combined indicators by regions (circle [•]: Region 1 [Java and Bali], up triangle [▲]: Region 2 [Sumatra], down triangle [▼]: Region 3 [Kalimantan, Sulawesi, Nusa Tenggara Barat, and Nusa Tenggara Timur], and square [▪]: Region 4 [Maluku and Papua]). Types of health-care facilities include primary health centers (PHCs) (*N* = 8,831), auxiliary PHCs (*N* = 22,853), village health posts (*N* = 28,692), and village maternity clinics (*N* = 14,396). The combined WatSan indicator is defined as a facility with access to basic water and sanitation services. The combined WatSan and Hygiene indicator is defined as a facility with access to basic water, sanitation, and hygiene (in a general consultation room) services. The combined WatSan, Hygiene, and Waste indicator is defined as a facility with access to basic water, sanitation, hygiene (in a general consultation room), and medical waste management services.

**Figure 3.**
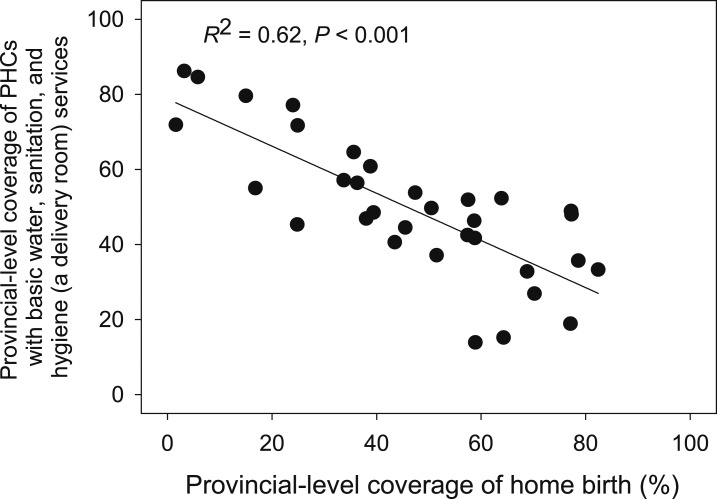
Relationship between provincial-level coverage of primary health centers (PHCs) with basic “WatSan and Hygiene (a delivery room)” services and provincial-level coverage of home birth. Only PHCs with a delivery room were included (*N* = 3,097). The WatSan and Hygiene (a delivery room) combined indicator is defined as a facility with access to basic water, sanitation, and hygiene (in a delivery room) services. Each dot represents one province (*N* = 33).

This study has several limitations. First, our basic WASH service figures are likely to be overestimated, particularly for sanitation. The WHO/UNICEF JMP service definition includes separated toilets for patients and staff as well as for women, provision of menstrual hygiene facilities and meeting the needs of people with limited mobility. These are critical to minimize risk of infection between staff and patients and to provide privacy and dignity for girls and women. However, this study could not include these components because of a lack of data on these indicators. A recent survey conducted by UNICEF in 2016 reported that most toilets (68%, *N* of PHCs = 28) were not sex-separated in PHCs in rural eastern Indonesia, indicating the challenges in PHCs to meet the basic sanitation service defined by the WHO/UNICEF JMP.^22^ These definitions, however, may need to be tailored to the country-context and HCF types; it would be unrealistic to provide several toilets in a village health post, for instance. Country-level discussions with government and stakeholders will be important to define and agree on service ladders and indicators in this respect. Second, national data were collected in 2010 and 2011 which may not reflect the current status of WASH services in HCFs. However, recent UNICEF survey data (2016) highlighted that only 39% (total *N* = 28) of surveyed rural PHCs in selected districts across three provinces had handwashing stations with water and soap at all points of care,^22^ indicating a major improvement may not yet have taken place in rural eastern Indonesia. Last, the presence of handwashing facilities does not necessarily reflect health staff compliance of handwashing behavior.

The study identified key recommendations for the SDG global monitoring of WASH in HCFs in LMICs. First, realistic basic services, in particular for sanitation, need to be defined based on HCF types. Current services ladders might be too ambitious for lower health service level HCFs. Setting minimum basic services (e.g., access to at least basic sanitation) depending on HCF types would help local government achieve the SDG goal in meaningful and realistic way. Second, the hand hygiene indicator could be more specific, rather than indicating “at points of care and toilets.” Our study showed the proportion of PHCs with handwashing access in all three rooms drastically dropped, whereas making collective actions difficult because of lack of detailed information. Prioritizing rooms that need cleaner environment most such as a delivery room could be strategic to reduce potential health risks effectively, whereas advanced service level may pursue access in all points of care.

In conclusion, this study produced the SDG baseline estimate of WASH services in HCFs in Indonesia, by adopting and contextualizing the emerging WHO/UNICEF JMP service ladder framework to existing national HCF census data sets. The findings suggest national systems need to be urgently strengthened for improving monitoring and WASH service delivery mechanisms in HCFs in Indonesia. Enhancing the use of the monitoring data with a feedback mechanism, strategic and efficient resource allocations to unserved regions/communities may help to minimize regional disparities. In response to these challenges, MoH, Indonesia, initiated a series of discussions on WASH in HCFs in 2017. These cross-department technical meetings aimed at 1) reviewing current policies, 2) exploring ways of strengthening existing national monitoring systems for tracking the progress of the SDG targets guided by SDGs’ definitions proposed by the WHO/UNICEF JMP; more concretely, strengthening a regular program monitoring system, known as ASPAK, managed by health-care facility unit of MoH would help district health office take actions including effective resource allocations and capacity building of health staff, whereas HCFs census by National Institutes of Health Research and Development, MoH (i.e., Rifaskes) every 5–10 years could validate the progress against the SDG targets, 3) agreeing key harmonized indicators to be monitored, and 4) exploring the potential of strengthening health facility accreditation mechanisms for incentivizing managers of HCF for actions.^23^ These efforts will be expected to provide a more up-to-date picture of WASH service conditions in HCFs in coming years and help inform planning to achieve universal access to basic WASH services in HCFs in Indonesia. Finally, further research is needed to better understand the linkage between WASH services and quality of health-care services within broader health systems strengthening to achieve effective UHC and promote people-centered care.

## Supplementary Material

Supplemental Material and Table
