# Supplementary material for: Water, Sanitation, and Hygiene Services in Public Health-Care Facilities in Indonesia: Adoption of World Health Organization/United Nations Children’s Fund Service Ladders to National Data Sets for a Sustainable Development Goal Baseline Assessment
Source: Am J Trop Med Hyg. 2018 Jun 25;99(2):546–51. doi: 10.4269/ajtmh.18-0044 (PMC6090366; doi:10.4269/ajtmh.18-0044)
Supplement: Supplementary file 1 [file tpmd180044.SD1.pdf]

Supplemental Material

Title: Water, sanitation and hygiene services in public healthcare facilities in Indonesia: Adoption of WHO/UNICEF service ladders to national data sets for a Sustainable Development Goal baseline assessment

Authors: Mitsunori Odagiri[1], Cahyorini[2], Khadijah Azhar[2], Aidan A. Cronin[1], Yulian Gressando[1], Indah Hidayat[3], Widya Utami[3], Karina Widowati[4], Airin Roshita[5], Rooswanti Soeharno[4], Sonny Priajaya Warouw[3], Ardhiantie[6]

[1]Water, Hygiene and Sanitation (WASH) Section, UNICEF Indonesia, [2]National Institute of Health Research and Development, Ministry of Health, Indonesia, [3]Environmental Health Unit, Ministry of Health, Indonesia, [4]Health Section, UNICEF Indonesia, [5]Nutrition Section, UNICEF Indonesia, [6]Community Health and Nutrition Unit, National Development Planning Agency (Bappenas), Indonesia

1. Questionnaires

The healthcare facility research questionnaire in Bahasa Indonesia is available at:

http://labdata.litbang.depkes.go.id/images/download/kuesioner/RIFAS/2011/pkm/KUESIONER.pdf

The village potential data questionnaire is available at:

http://microdata.worldbank.org/index.php/catalog/1826

Table S1. Water, Sanitation, Hygiene and Medical Waste services in four types of healthcare facilities

Service types	Service levels	Rifaskes (2011)			PODES (2011)			
		PHCs[d] (n = 8,831)	Urban (n = 2,322)	Rural (n = 6,509)	PHCs (n = 9,210)	Auxiliary PHCs (n = 22,853)	Village health posts (n = 28,692)	Village maternity clinics (n = 14,396)
Water	Basic services	82.3%	89.9%	79.6%	87.0%	60.5%	57.2%	56.6%
	Limited services	14.9%	7.9%	17.5%	5.4%	12.1%	10.9%	10.7%
	No services	2.8%	2.2%	3.0%	7.6%	27.3%	31.9%	32.7%
Sanitation	Basic services	88.1%	94.7%	85.8%	89.5%	63.0%	57.4%	55.1%
	Limited services	8.7%	3.5%	10.6%	4.3%	15.4%	6.8%	6.5%
	No services	3.1%	1.8%	3.6%	6.2%	21.5%	35.8%	38.4%
Hygiene (n = 8,831)	Basic/Limited services	53.5%	66.0%	49.0%	-	-	-	-
(General consultation room)	No services	46.5%	34.0%	51.0%	-	-	-	-
Hygiene (n = 5,628)	Basic/Limited services	32.5%	44.6%	28.3%	-	-	-	-
(Immunization room)	No services	67.5%	55.4%	71.7%	-	-	-	-
Hygiene (n = 3,097)	Basic/Limited services	66.5%	74.5%	64.8%	-	-	-	-
(Delivery room)	No services	33.5%	25.5%	35.2%	-	-	-	-
Medical waste	Basic services	35.1%	60.9%	20.2%	-	-	-	-
	Limited services	34.0%	21.2%	38.5%	-	-	-	-
	No services	30.9%	17.9%	41.3%	-	-	-	-
WatSan[a]	Basic services	76.4%	87.1%	72.6%	82.3%	49.1%	46.2%	45.1%
WatSan and Hygiene[b]	Basic services	46.2%	60.2%	41.2%	-	-	-	-
WatSan, Hygiene and Waste[c]	Basic services	20.2%	41.9%	12.5%	-	-	-	-

WatSan[a]: a combined indicator defined as a facility with access to basic water and sanitation services, WatSan and Hygiene[b]: a combined indicator defined as a facility with access to basic water, sanitation and hygiene (in a general consultation room) services, WatSan, Hygiene and Waste[c]: a combined indicator defined as a facility with access to basic water, sanitation, hygiene (in a general consultation room) and medical waste management services, PHCs[d]: primary health centers.
